# P2X receptors trigger intracellular alkalization in isolated perfused mouse medullary thick ascending limb

**DOI:** 10.1111/apha.12417

**Published:** 2014-11-24

**Authors:** P I A de Bruijn, M Bleich, H A Praetorius, J Leipziger

**Affiliations:** 1 Department of Biomedicine, Physiology and Biophysics, Aarhus UniversityAarhus C, Denmark; 2 Institute of Physiology, Christian-Albrechts-UniversityKiel, Germany

**Keywords:** intracellular pH, loop of Henle, Na^+^/H^+^ exchanger 3, P2 receptors, purinergic

## Abstract

**Aims:**

Extracellular ATP is an important regulator of renal tubular transport. Recently, we found that basolateral ATP markedly inhibits Na^+^ and Cl^−^ absorption in mouse medullary thick ascending limb (mTAL) via a P2X receptor. The underlying mechanism that mediates this ATP-dependent transport inhibition in mTAL is, however, unclear. The renal outer medullary K^+^ channel (ROMK) is sensitive to intracellular pH where a reduction leads to closing of ROMK. We speculated that P2X receptor stimulation in the TAL could lead to changes in pH_i_, leading to a reduction in NaCl transport.

**Methods:**

To test this hypothesis, we measured pH_i_ in single perfused mouse mTALs using the fluorescent ratiometric dye 2′,7′-bis-(2-carboxyethyl)-5-(and-6)-carboxyfluorescein acetoxymethylester.

**Results:**

Interestingly, basolateral ATP (100 *μ*m) caused a prominent, reversible intracellular alkalization of mTAL, with an average pH_i_ increase of 0.14 ± 0.02 (*n* = 14). This was completely abolished by the P2X receptor antagonist periodate-oxidized ATP (50 *μ*m). The P2X receptor-mediated intracellular alkalization required the activity of the apical Na^+^/H^+^ exchanger (NHE3). Typically, G_q_-coupled receptors cause a significant acidification of tubular epithelial cells, which was confirmed in this study, by P2Y_2_ and Ca^2+^ sensing receptor stimulation.

**Conclusion:**

This study reports that stimulation of basolateral P2X receptors causes a substantial intracellular alkalization in the isolated perfused mouse mTAL. This intracellular alkalization is mediated through an increased apical NHE3 activity, similar to what we previously observed when tubular transport is inhibited with furosemide. This increased NHE3 activity causes H^+^ secretion in the mTAL and provides further support that the TAL is a site of urinary acidification.

The thick ascending limb of Henle's loop (TAL) is essential for overall body water and salt homoeostasis. The TAL reabsorbs 20–30% of the filtered NaCl load and creates the osmotic gradient in the renal medulla, thereby facilitating H_2_O reabsorption in the collecting duct. Na^+^ uptake in the TAL is mediated by the Na^+^ K^+^ Cl^−^ cotransporter NKCC2 (Greger 1985). Na^+^ leaves the TAL through the basolateral Na^+^/K^+^ ATPase (Greger 1985), and Cl^−^ exits via the basolateral Cl^−^ channel ClC_kb_ (Greger 1985). K^+^ is partially recycled into the lumen by the renal outer medullary K^+^ channel (ROMK). Inhibition of ROMK with 3 mm Ba^2+^ results in a complete block of Na^+^ and Cl^−^ reabsorption in TAL (Greger & Schlatter 1983). ROMK has been shown to be sensitive to intracellular pH (pH_i_), where a minor cytosolic acidification results in closing of the channel (Bleich *et al*. 1990, Choe *et al*. 1997, Leipziger *et al*. 2000). It has never been demonstrated whether the unique pH_i_ dependence of ROMK serves the function of regulating transport rates in the TAL, that is if agonist- or antagonist-induced changes of pH_i_ actually alter Na^+^ and Cl^−^ absorption.

The autocrine and paracrine signalling pathway through purinergic (P2) receptors plays a role in the regulation of renal epithelial transport. The P2 receptors are subdivided into G-protein-coupled receptors (P2Y) and ligand-gated ion channels (P2X). Both receptor types are abundantly expressed along the nephron and are generally involved in inhibition of transport processes (Kishore *et al*. 1995, Lehrmann *et al*. 2002, Bailey 2004, Rieg *et al*. 2007, Pochynyuk *et al*. 2008). The TAL has been shown to express functional apical P2Y_2_ receptors and basolateral P2Y_2_ and P2X receptors. Stimulation of the P2 receptors of either type results in rises in [Ca^2+^]_i_ (Jensen *et al*. 2007, Geyti *et al*. 2008), and extracellular ATP is known to reduce O_2_ consumption in TAL suspensions, which is the likely result of Na^+^ and Cl^−^ transport inhibition (Silva & Garvin 2009). Indeed, transport measurements in isolated perfused medullary TAL (mTAL) show that basolateral ATP causes an inhibition of Na^+^ and Cl^−^ transport that is mediated through multiple P2X receptors, including the P2X_4_ receptor (Marques *et al*. 2012). The mechanism for this transport inhibition is not yet established. In this study, we wanted to investigate whether extracellular ATP induces changes in pH_i_ in isolated perfused mouse mTAL. It was speculated that stimulation of P2X receptors could cause an intracellular acidification, which potentially could trigger transport inhibition. In several studies that use agonists to elevate [Ca^2+^]_i_, it has been established that a rise in [Ca^2+^]_i_ is associated with intracellular acidification (Berk *et al*. 1987, Sage *et al*. 1990). This is also the case for P2X receptors, where stimulation associates with an intracellular acidification (Henriksen & Novak 2003). Thus, P2X_4_ receptor stimulation could potentially result in a decrease in cytosolic pH, thereby closing ROMK to cause an inhibition of Na^+^ and Cl^−^ transport in the TAL.

This hypothesis was proven wrong and, surprisingly, basolateral ATP caused a significant, sustained and reversible intracellular alkalization through P2X receptor stimulation in perfused mouse mTALs. The ATP-induced increase in pH_i_ is mediated by the apical Na^+^/H^+^ exchanger 3 (NHE3) and can only be observed in an actively transporting tubule. Our data imply that ATP triggers NHE3-dependent H^+^ secretion through an inhibition of tubular transport. The results also reflect a potential role of the TAL in urinary acid secretion.

## Materials and methods

### Tubule perfusion

All mouse handling of animals complied with Danish animal welfare regulations. Animals had free access to standard rodent diet and tap water. Experiments were performed on 4- to 6-week-old mice with a mixed genetic background (B6D2/SV129). Mice were sacrificed by cervical dislocation, the kidneys collected, placed in ice-cold control solution containing (in mm) 145 NaCl, 0.4 KH_2_PO_4_, 1.6 K_2_HPO_4_, 5 d-glucose, 1 MgCl_2_, 1.3 Ca-gluconate and 5 *N*-2-hydroxyethylpiperazine-*N*-2-ethane-sulfonic acid (HEPES) and were subsequently sliced. The slices were placed in a dissection chamber with cold (4 °C) control solution. mTALs were isolated from the inner stripe of the outer medulla (ISOM) with ultrafine forceps. The dissected mTALs were transferred to a perfusion chamber mounted on an inverted microscope (Axiovert 100 TV; Zeiss, Jena, Germany) and perfused with a concentric pipette system as described previously (Greger & Hampel 1981). TALs were stabilized on the bath bottom with a holding pipette. Tubules were bathed and perfused with control solution from one side with the tubule outflow left open. All experiments were performed at 37 °C, and agonist and antagonist solutions were prepared fresh.

### Fluorescence recording

The set-up for fluorescence microscopy consisted of an inverted microscope with a 63× C-Apochromat 1.2 water (Zeiss) objective, a VisiChrome polychromator system (Visitron, Puchheim, Germany) and a digital CCD camera (Spot pursuit 1.4 monochrome; Diagnostic Instruments, Sterling Heights, MI, USA). Images were acquired, and data analysed with standard software (visiview; Visitron). Intracellular pH was measured with the ratiometric fluorescent dye 2′,7′-bis-(2-carboxyethyl)-5-(and-6)-carboxyfluorescein acetoxymethylester (BCECF AM; Invitrogen, Carlsbad, CA, USA). Tubules were incubated with 5 *μ*m basolateral BCECF AM in control solution for 20 min at RT during continuous perfusion with control solution, followed by a 5-min washout period. The pH_i_ was measured as the emission ratio at 490/436 nm excitation during 5-s intervals. To reduce photo damage to the tissue, the excitation speed was 50 ms at 436 nm and 25 ms at 490 nm. A 500-nm beam splitter and a 520/560 band pass were used. Experimental manipulations were carried out after a stable fluorescence signal was achieved, and fluorescence of the entire tubule was recorded for analysis. ATP, oxidized ATP (oATP) (Sigma-Aldrich) and #4167 (kindly provided by Sanofi Aventis, Germany) were freshly dissolved in H_2_O prior experiments. Furosemide was dissolved in DMSO to 0.1 m stock solution and dissolved further in H_2_O prior to each experiment.

Calibration of the BCECF signal was performed using the high K^+^ and nigericin method (Thomas *et al*. 1979) in a paired fashion. The calibration solution was used previously in similar experiments (Watts & Good 1994, Odgaard *et al*. 2004) and contained (in mm) 95 KCl, 15 NaCl, 0.4 NaH_2_PO_4_, 1.6 Na_2_HPO_4_, 5 glucose, 1 MgCl_2_, 1.3 Ca-gluconate, 25 HEPES and 20 N-Methyl-D-glucamine, supplemented with 2 *μ*m nigericin. The solution was set at pH 6.5, 7.4 and 7.8.

### Statistics

All data are presented as mean ± SEM in all series, *n* indicates the number of tubules used. For each series, no more than two tubules were used from a single mouse. Data were tested for normality with the Kolmogorov–Smirnov test. Differences between experimental conditions were analysed using the paired or unpaired Student's *t*-test or anova where necessary. In all cases, *P* < 0.05 was considered significant.

## Results

### Basolateral ATP induces an alkalization in mTAL cells

**Figure 1:**
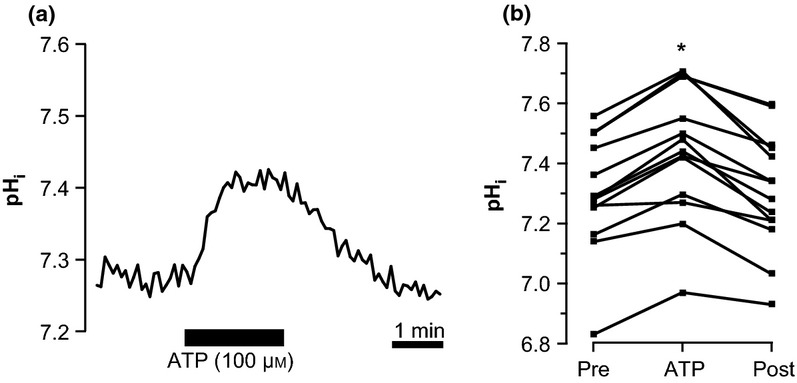
Basolateral ATP causes an intracellular alkalization in perfused medullary thick ascending limb (mTAL). (a) Representative trace of a perfused mTAL loaded with 2′,7′-bis-(2-carboxyethyl)-5-(and-6)-carboxyfluorescein acetoxymethylester (BCECF AM). ATP (100 *μ*m) is applied to the bath for 2 min. (b) Summarized data of the experiments with 100 *μ*m basolateral ATP, *n* = 14. *Indicates statistical significance (*P* < 0.001).

**Figure 2:**
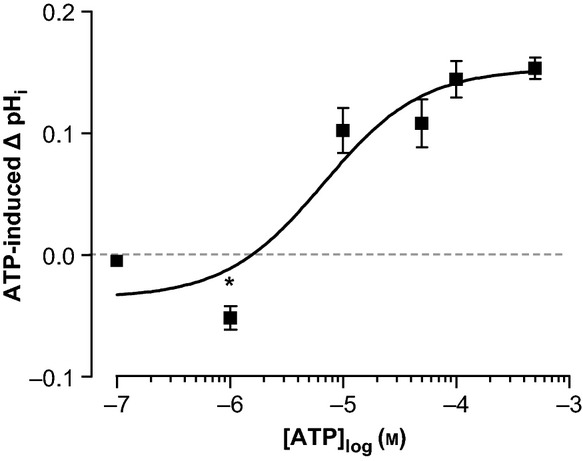
Concentration–response curve of basolateral ATP-induced alkalization in perfused medullary thick ascending limbs, *n* = 6–14. Values are read after 2-min ATP exposure. *Indicates statistical significance.

Figure1a shows a typical pH_i_ recording of a perfused mTAL. Under resting conditions, the pH_i_ of this tubule was 7.28. The average pH_i_ of the summarized data from all perfused mTALs was 7.31 ± 0.05 (*n* = 14, Fig.1b). Application of basolateral ATP (100 *μ*m) caused a reversible intracellular alkalization in perfused mTAL, which was sustained during the 2-min exposure to ATP and then returned to the basal pH_i_ after a washout period of 2–5 min. The average pH_i_ during ATP exposure was 7.45 ± 0.06, and after 5-min washout, the pH_i_ recovered to 7.31 ± 0.05 (Fig.1b, *P* < 0.0001). These data reflect that ATP causes an intracellular alkalization with a magnitude of 0.14 ± 0.02 pH units. Figure2 shows the concentration–response curve of ATP in a range from 100 nm to 500 *μ*m. The estimated EC_50_ was 6.74 ± 1.67 *μ*m. Interestingly, low concentrations of ATP (1 *μ*m) resulted in a decrease in pH_i_, suggesting that basolateral ATP has a dual effect on intracellular pH in mouse mTAL.

### P2Y_2_ receptor stimulation causes an intracellular acidification

**Figure 3:**
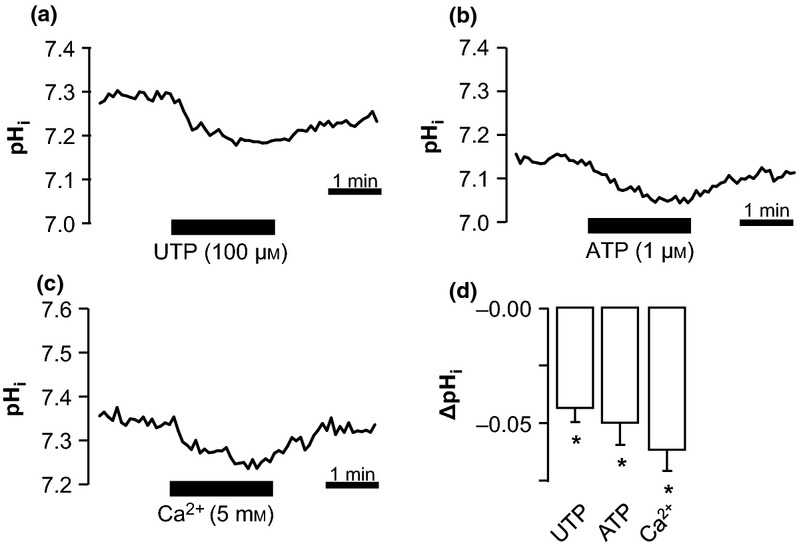
P2Y_2_ and Ca^2+^ sensing receptors (CaSR) stimulation causes an acidification in perfused medullary thick ascending limbs (mTALs). (a–c) Representative traces of perfused mTALs exposed for 2 min to basolateral UTP (100 *μ*m), ATP (1 *μ*m) and high Ca^2+^ (5 mm). (d) Summarized data of ΔpH_i_ induced with UTP, ATP and Ca^2+^ (*n* = 4–8). *Indicates statistical significance *P* < 0.01.

Our group previously demonstrated that of the P2Y receptor family, only P2Y_2_ and P2Y_6_ are expressed in murine mTAL (Marques *et al*. 2012). To investigate the role of P2Y receptors in the ATP-induced alkalization, we used the potent P2Y_2_ receptor agonist UTP that does not activate P2X receptors. Figure3a shows a typical experiment of a perfused mTAL exposed to basolateral UTP (100 *μ*m), causing a small but significant acidification (ΔpH_i_ 0.04 ± 0.01, *n* = 7, *P* < 0.001). UDP, a specific P2Y_6_ receptor agonist, did not cause any changes in pH_i_ (results not shown). The magnitude of the UTP-induced acidification was similar to that seen when 1-*μ*M ATP was applied to the basolateral side (ΔpH_i_ 0.05 ± 0.01, *n* = 6, Fig.3b), which is sufficient to stimulate the P2Y_2_ receptor (Abbracchio *et al*. 2006). Stimulation of the Ca^2+^ sensing receptors (CaSR) with increased extracellular [Ca^2+^] (5 mm) also acidified perfused mTALs. These findings indicate that stimulation of Gq-protein-coupled receptors such as P2Y_2_ and CaSR in the TAL causes a small intracellular acidification.

### The ATP-induced alkalization is mediated through P2X receptors

**Figure 4:**
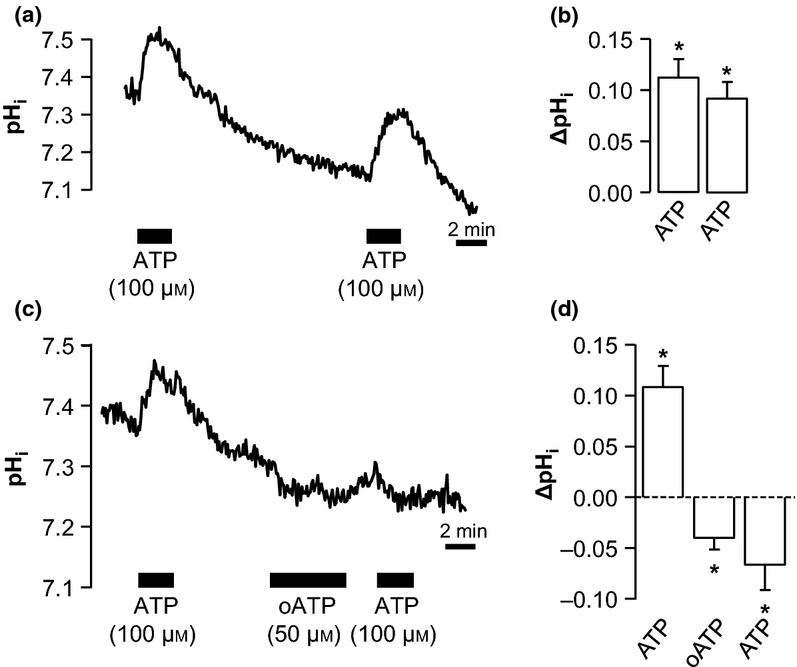
P2X receptor antagonist oxidized ATP (oATP) inhibits the ATP-induced alkalization. (a+b) Original trace and summary (ΔpH_i_) of time control experiments, where a perfused medullary thick ascending limb is exposed to basolateral ATP twice with a 12-min washout period in between. (c+d) Original trace and summary (ΔpH_i_) of experiments, where pre-incubation with oATP (50 *μ*m, 5-min exposure) completely inhibits the ATP-induced alkalization. *Indicates statistical significance *P* < 0.01, *n* = 6.

We previously demonstrated that P2X_1_, P2X_4_ and P2X_5_ receptors are expressed in TAL of mice (Marques *et al*. 2012). To confirm that the ATP-induced alkalization is mediated by P2X receptor stimulation, we used the unspecific irreversible P2X receptor antagonist oATP. Figure4a shows an original trace of a time control experiment, for two consecutive applications of basolateral ATP to a perfused mTAL separated by 12-min washout. It is clear from both Figure4a and the summarized data in Figure4b that ATP induced an alkalization of comparable size in both cases. When, however, ATP was applied after the irreversible P2X receptor antagonist oATP (50 *μ*m), the ATP-induced alkalization was completely abolished (Fig.4c). Instead, an acidification was observed, congruent with a residual P2Y_2_ receptor stimulation (Fig.4d). Thus, the ATP-induced alkalization is mediated through basolateral P2X receptors.

### ATP-induced alkalization is mediated through activation of apical NHE3

**Figure 5:**
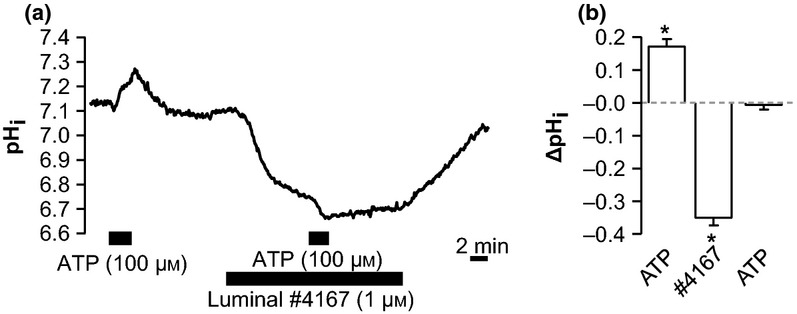
Effect of Na^+^/H^+^ exchanger (NHE3) inhibition with #4167 on ATP-induced alkalization. (a) Original experiment of perfused medullary thick ascending limb exposed to luminal #4167 (1 *μ*m) and basolateral ATP (100 *μ*m). (b) Summary (ΔpH_i_) of the series. *Indicates statistical significance *P* < 0.01, *n* = 8.

A recent study from our group has established that furosemide causes an intracellular alkalization in the mTAL through increased apical NHE3-mediated H^+^ secretion (de Bruijn *et al*. 2013). To investigate whether the ATP-induced intracellular alkalization occurs by the same mechanism, basolateral ATP was tested in the presence of the specific NHE3 blocker #4167 (Reuter *et al*. 2008). Figure5a shows that luminal #4167 (1 *μ*m) caused a significant intracellular acidification (ΔpH −0.35 ± 0.02, *n* = 8, Fig.5b). During this NHE3 inhibition, the ATP-induced alkalization was completely abolished in five of eight experiments, whereas in three experiments, the alkalization was strongly attenuated. These data indicate that apical NHE3 activity is required for the ATP-induced alkalization.

### ATP-induced alkalization is abolished in the presence of furosemide

**Figure 6:**
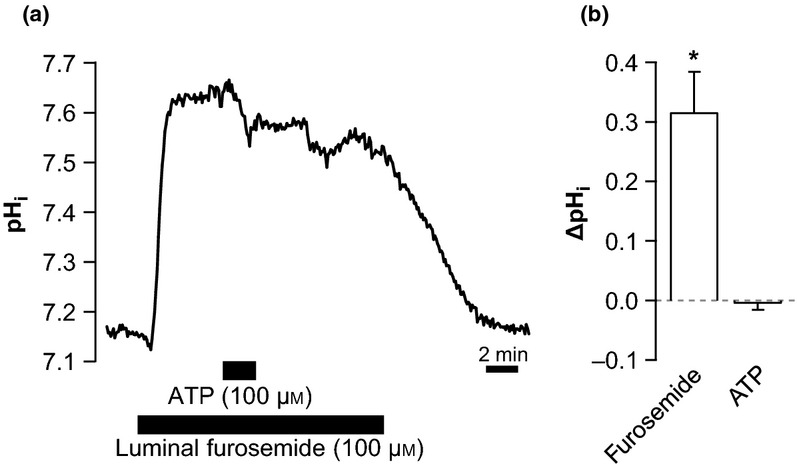
Luminal furosemide inhibits the basolateral ATP-induced alkalization. (a) Original trace of an experiment, where ATP (100 *μ*m) is added to the bath in the presence of luminal furosemide (100 *μ*m). (b) Summary (ΔpH_i_) of the data, *n* = 6. *Indicates statistical significance, *P* < 0.001.

Furosemide and ATP both cause significant intracellular alkalizations in mTAL that require NHE3 activity (this study and de Bruijn *et al*. 2013), and they are known to reduce tubular transport in this segment (Greger 1985, Marques *et al*. 2012). It was therefore interesting to study whether the pH_i_ effects of the two substances are additive. Figure6a illustrates that furosemide causes a marked intracellular alkalization from pH 7.25 ± 0.08 to pH 7.60 ± 0.02 (*n* = 6, Fig.6b). When basolateral ATP (100 *μ*m) was added during the continuous presence of luminal furosemide, pH_i_ remained unchanged. These results do not support an additive effect of these two alkalizing stimuli and thus are consistent with the notion of a common underlying cause.

## Discussion

In this study, we describe the surprising finding that basolateral P2X receptor stimulation triggers a marked alkalization in the isolated perfused mouse mTAL. It is well established that GPCRs and ligand-gated ion channels in a variety of tissues trigger a significant intracellular acidification (Berk *et al*. 1987, Sage *et al*. 1990, Henriksen & Novak 2003). We were therefore curious to define the mechanism for the pronounced ATP-triggered alkalization. As a first step, we confirmed that P2Y_2_ receptor and CaSR stimulation triggered the expected acidification in the mTAL. We further showed that the ATP-stimulated alkalization occurred via basolateral P2X receptors. As the experiments were conducted in HEPES buffer, the ATP-stimulated alkalization must reflect the removal of protons from the cytosol. Finally, we demonstrated that the P2X receptor-mediated alkalization was completely inhibited by blocking the apical NHE3. In summary, our results are consistent with an ATP-activated H^+^ secretion into the tubular lumen via the Na^+^-dependent H^+^ exchanger NHE3 in mTAL from mice.

Clues to a mechanism of how ATP activates NHE3 can be found in a parallel report from our group (de Bruijn *et al*. 2013). In this work, we demonstrated that furosemide causes a prominent intracellular alkalization, which results from a sudden drop in [Na^+^]_i_ that apparently leads to an increased activity of the apical NHE3 by a more favourable chemical Na^+^ gradient. The marked drop of [Na^+^]_i_ is best explained by acute inhibition of Na^+^ influx via the apical NKCC2 cotransporter during continuous basolateral Na^+^ efflux via the Na^+^/K^+^ ATPase. Moreover, it was shown that furosemide stimulated H^+^ secretion into the mTAL lumen by directly measuring luminal pH. One main conclusion of this parallel work was that transport inhibition with loop diuretics triggers a marked H^+^ secretion and therefore urinary acidification.

In this study, we identify that basolateral P2X receptor stimulation leads to the activation of the apical NHE3. However, the cellular mechanism could not be resolved. It is well established that NHEs are activated by cell shrinkage and thus involved in regulatory volume increase (RVI, Hoffmann *et al*. 2009). However, NHE3 has been demonstrated to be activated by cell swelling rather than shrinkage in rat TAL (Watts & Good 1999, Good *et al*. 2000). Activation of the P2X receptors results in the influx of cations and, therefore, could result in cell swelling. Preliminary data confirm that ATP causes cell volume increases in perfused mTAL and show that hyposmolality result in an intracellular alkalization (unpublished data). It is thus likely that the observed intracellular alkalization caused by P2X receptor stimulation is a consequence of cell swelling-induced NHE3 activation. However, Watts and Good have shown that this swelling-induced activation of NHE3 still takes place in the presence of furosemide (Watts & Good 1999). Our data with furosemide and ATP show that NHE3 can apparently not be further stimulated by P2X receptor activation (Fig.6). More detailed studies on the activation of NHE3 in both cases are required to fully understand its regulation.

**Figure 7:**
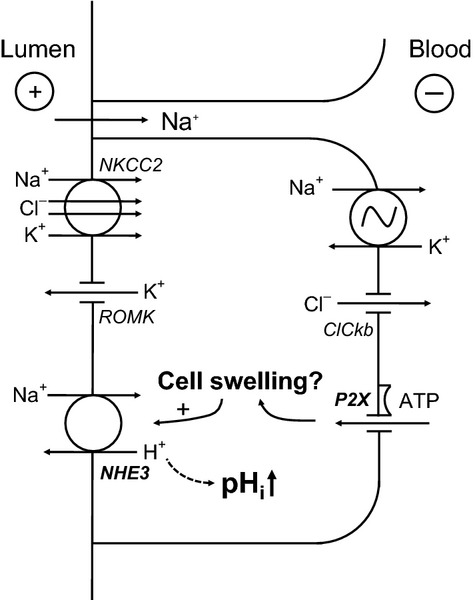
Model of P2X receptor-mediated intracellular alkalization in medullary thick ascending limb. Stimulation of the P2X receptor causes an influx of cations, which should lead to cell swelling. This in turn may stimulate the apical Na^+^/H^+^ exchanger (NHE3), leading to the observed intracellular alkalization.

Basolaterally applied ATP is established to inhibit Na^+^ and Cl^−^ absorption substantially (approx. 25%) via P2X receptors (Marques *et al*. 2012). The current results indicate that inhibition of transport, irrespective of the mode of induction, associates with an intracellular alkalization caused by increased H^+^ secretion. Taken together, these results indicate that ATP, much similar to furosemide, increases the driving force for luminal H^+^ exit via the NHE3 (Fig.7 for model). It is worth to note that partial transport inhibition as seen under P2X receptor stimulation causes a moderate alkalization as compared to a massive pH effect when Na^+^ and Cl^−^ absorption was fully inhibited with furosemide. These results indicate that the rate of Na^+^ and Cl^−^ absorption inversely correlates with the rate of H^+^ secretion via apical NHE3. Indeed, it has been shown that AVP, which stimulates NKCC2 activity (Welker *et al*. 2008, Marques *et al*. 2013), reduces 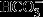 reabsorption, consistent with a decrease in NHE3 activity (Good 1990).

The main motivation for this study was to investigate the underlying signalling mechanism that explains how basolateral P2X receptor stimulation inhibits Na^+^ and Cl^−^ transport in the TAL. Currently, it was unresolved whether alterations of pH_i_ could matter in this signalling cascade. The current through the ROMK channel is known to be significantly reduced by intracellular acidification, which potentially could lead to transport inhibition, much similar to that observed with luminal Ba^2+^. Clearly, our results show that P2X receptor stimulation does not cause an intracellular acidification and therefore argues against this hypothesis.

Several experimental observations argue against a critical role of intracellular acidification as important modulator of Na^+^ and Cl^−^ absorption in the TAL. We show here that P2Y_2_ receptor and CaSR stimulation resulted in a small intracellular acidification. It is, however, unlikely that this acidification results in ROMK closure and transport inhibition, as it has been shown that acute basolateral P2Y_2_ receptor stimulation has no effect on Na^+^ and Cl^−^ reabsorption in the TAL (Marques *et al*. 2013). In accordance with this, inhibition of apical Na^+^/H^+^ exchange with amiloride, which is known to cause marked intracellular acidifications in the TAL, does not change the transepithelial voltage in perfused mouse and rat mTAL (Good 1985). These results again suggest that the pH_i_ sensitivity of ROMK may not play an important role in regulation of the transepithelial transport in TAL.

In summary, this study reports the novel finding that stimulation of basolateral P2X receptors causes a substantial intracellular alkalization in the isolated perfused mouse mTAL. Together with previous studies (Good 1990, de Bruijn *et al*. 2013), the cellular mechanism is described and highlights that the intracellular alkalization is mediated through an increased apical NHE3 activity. This increased NHE3 activity causes H^+^ secretion in the mTAL and provides further support that the TAL is a site of urinary acidification.

## Conflicts of interest

None.

## 

We greatly appreciate the expert technical assistance from Edith B. Møller and Helle Jakobsen. Funding was provided by the Danish Medical Research Council.
